# Chemically Defined Diet Alters the Protective Properties of Fructo-Oligosaccharides and Isomalto-Oligosaccharides in HLA-B27 Transgenic Rats

**DOI:** 10.1371/journal.pone.0111717

**Published:** 2014-11-04

**Authors:** Petya Koleva, Ali Ketabi, Rosica Valcheva, Michael G. Gänzle, Levinus A. Dieleman

**Affiliations:** 1 Department of Agricultural, Food and Nutritional Science, University of Alberta, Edmonton, Canada; 2 Centre of Excellence for Gastrointestinal Inflammation and Immunity Research, University of Alberta, Edmonton, Canada; 3 School of Food and Pharmaceutical Engineering, Hubei University of Technology, Wuhan, China; Institut Pasteur de Lille, France

## Abstract

Non-digestible oligosaccharides (NDO) were shown to reduce inflammation in experimental colitis, but it remains unclear whether microbiota changes mediate their colitis-modulating effects. This study assessed intestinal microbiota and intestinal inflammation after feeding chemically defined AIN-76A or rat chow diets, with or without supplementation with 8 g/kg body weight of fructo-oligosaccharides (FOS) or isomalto-oligosaccharides (IMO). The study used HLA-B27 transgenic rats, a validated model of inflammatory bowel disease (IBD), in a factorial design with 6 treatment groups. Intestinal inflammation and intestinal microbiota were analysed after 12 weeks of treatment. FOS and IMO reduced colitis in animals fed rat chow, but exhibited no anti-inflammatory effect when added to AIN-76A diets. Both NDO induced specific but divergent microbiota changes. Bifidobacteria and *Enterobacteriaceae* were stimulated by FOS, whereas copy numbers of *Clostridium* cluster IV were decreased. In addition, higher concentrations of total short-chain fatty acids (SCFA) were observed in cecal contents of rats on rat chow compared to the chemically defined diet. AIN-76A increased the relative proportions of propionate, iso-butyrate, valerate and iso-valerate irrespective of the oligosaccharide treatment. The SCFA composition, particularly the relative concentration of iso-butyrate, valerate and iso-valerate, was associated (P≤0.004 and r≥0.4) with increased colitis and IL-1 β concentration of the cecal mucosa. This study demonstrated that the protective effects of fibres on colitis development depend on the diet. Although diets modified specific cecal microbiota, our study indicates that these changes were not associated with colitis reduction. Intestinal inflammation was positively correlated to protein fermentation and negatively correlated with carbohydrate fermentation in the large intestine.

## Introduction

Ulcerative colitis (UC) and Crohn's disease (CD), collectively called chronic inflammatory bowel diseases (IBD), are characterized by chronic inflammation of the gut. Intestinal bacteria contribute to initiation and perpetuation of chronic intestinal inflammation [1]. IBD patients exhibit a lower diversity of intestinal bacteria, indicating microbial imbalance or ‘dysbiosis’ [2], [3]. Inflammation in IBD patients generally occurs in those parts of the gastrointestinal tract with the highest abundance of microorganisms [4]. The composition and activity of intestinal microbiota is modulated by the diet [5] and the diet is also thought to play a role in IBD development and progression [6]. Diets high in sucrose, refined carbohydrates and ω-6 polyunsaturated fatty acids increase the risk of developing IBD [7], [8]. A ‘Western’ diet high in animal fat, proteins and refined carbohydrates, but low in fruits, vegetables and whole grains is associated with increased incidence and prevalence of Crohn's disease [9]. Conversely, dietary fibres, ω-3 polyunsaturated fatty acids or vitamin D attenuate chronic intestinal inflammation in animal studies [10], [11].

Non-digestible carbohydrates are emerging as potential therapy for IBD. Fructo-oligosaccharides (FOS) are among the best studied dietary compounds. FOS are indigestible linear fructosyl- β-(2→1)-(fructosyl)_n_-β-(2→1)-glucose oligomers [12]. FOS occur in several edible plants and are produced by *Lactobacillus* species in cereal fermentations [12], [13]. FOS stimulate growth of intestinal bifidobacteria in humans [12], [14]. β-Fructans reduced colitis in HLA B27 transgenic rats, a validated spontaneous IBD model [11], [15]. The protective effect of FOS was associated with an increased abundance of intestinal bifidobacteria [11], [15]. However, FOS did not attenuate inflammation in dextran sodium sulphate (DSS)-induced colitis in rats fed a purified diet [16] and supplementation of a non-purified diet with FOS reduced DSS-induced colitis in mice, whereas FOS supplementation of a purified diet exacerbated colitis [17]. Taken together, these studies suggest that the background diet affects FOS-mediated colitis reduction.

Comparable to the anti-inflammatory effect of FOS, resistant starch, lactulose, and isomalto-oligosaccharides (IMO) also attenuated intestinal inflammation in experimental models of IBD [18], [19], . The effect of these non-digestible carbohydrates on the composition and activity of intestinal microbiota overlaps only partially with the effect of FOS [21]. Particularly commercial IMO, which consist predominantly of α-(1→6) linked isomalto-oligosaccharides and α-(1→4) and α-(1→6) linked oligosaccharides of the panose-series [21], [22], stimulate growth of intestinal lactobacilli, but not of bifidobacteria in rodents [21]. Stimulation of intestinal lactobacilli and bifidobacteria by dietary IMO was observed in human studies [23], [24]. However, the effect of non-digestible carbohydrates other than FOS on intestinal microbiota in animal models for IBD remains poorly characterized [19], [20].

Despite the increasing body of evidence demonstrating beneficial effects of dietary fibre on intestinal inflammation in animal models of IBD, only few studies compare the effect of oligosaccharides that stimulate different members of intestinal microbiota. The present study aimed to compare the effects of two non-digestible oligosaccharides, FOS and IMO, which exert divergent effects on intestinal microbiota, and on reduction of colitis in HLA-B27 transgenic rats. To account for the interaction between oligosaccharides and the diet [17], [18], oligosaccharides were added to the chemically defined diet AIN-76A or to a standard rat chow. In order to investigate whether specific microbiota changes mediated the colitis-modulating effects of these fibres, we studied the composition of cecal and fecal microbiota, quantified metabolites of intestinal microbiota, and assessed chronic intestinal inflammation.

## Materials and Methods

### Animals, diets and study design

Animal use was approved by the Animal Care and Use Committee of the University of Alberta and conducted in accordance with the Canadian Council on Animal Care Guidelines. HLA-B27 transgenic rats, a validated animal model mimicking chronic inflammatory disease in humans [25], [26], were used in the study. Rats were housed two per cage in a temperature- (22°C) and light-controlled (12 h light/dark cycle) environment. Food and water were provided *ad libitum*. Animals were fed a commercial rat chow containing 20% protein, 11% fat, 34% starch and 15% sugars (5053 PicoLab Rodent Diet 20, Lab Diet Inc., Leduc, AB, Canada) or the chemically defined diet AIN-76A containing sucrose as the main carbohydrate source [27] (Teklad Custom Research Diet, Harland Laboratories, Madison, WI, USA) (see Tables S1 and S2 in File S1 for the detailed composition AIN-76A and rat chow diets, respectively). Rats were randomly allocated to six treatment groups: two control groups on rat chow or AIN-76A diet only; four treatment groups on rat chow or AIN-76A supplemented with 8 g/kg body weight of either FOS (Orafti P-95, Raffinerie Tirlemontoise, Tienen, Belgium); or IMO (Vitafibre, BioNeutra, Edmonton, Canada). Four females and four males were assigned to each group. The average feed consumption of the rats was 20 g/d. The body weight of rats was monitored every 2 weeks and the fibre content of the diet was adjusted to achieve an intake of 8 g/kg body weight and day, matching oligosaccharide levels that were previously reported to be protective without inducing adverse effects [15], [16], [17]. Treatment started at 4 weeks of age, before colitis occurred, and continued until 16 weeks of age [11]. Fecal samples were collected immediately after defecation from each group at 4 and 16 weeks of age. Animals were killed at 16 weeks by CO_2_ asphyxia. At necropsy, cecal and colonic tissues and digesta were collected for histological examination, quantification of mucosal IL-1β, as well as for microbiota analysis. Feces, tissue samples and cecal contents were immediately frozen and stored at −80°C.

### Histology and enzyme-linked immunosorbent assay (ELISA)

Histological damage of cecum and colon was scored using a validated scale ranging from zero to four as previously described [26]. Samples were scored blindly employing a validated microscopic inflammation scale [26] using light microscopy. Tissues were examined for the following parameters: (i) presence of epithelial cell exfoliation; (ii) crypt loss; (iii) mucosal thickening and (iv) submucosal cell infiltration. The observation of microscopic inflammation was confirmed by quantification of the mucosal interleukin-1β (IL-1β) as an additional, objective marker for colitis [15]. IL-1β was quantified in cecal and colonic tissue homogenates using a commercial rat-specific IL-1β ELISA DuoSet ELISA Development System kit (R&D Systems, Inc., Minneapolis, MN, U.S.A.).

### DNA isolation

Total DNA was isolated from feces and cecal contents with a QIAamp DNA Stool Mini Kit (Qiagen, Inc. Mississauga, ON, Canada). DNA concentration and quality were determined spectrophotometrically using a NanoDrop system ND-1000, version 3.3.0 (Thermo Fisher Scientific, Inc., Ottawa, ON, Canada). Samples were diluted prior to PCR analysis, in order to adjust DNA concentrations to 50 mg/L.

### Quantitative PCR (qPCR)

Intestinal microbiota were analysed by quantitative PCR using primers, PCR conditions and experimental protocols as described previously [11]. Butyrate-producing bacteria were enumerated based on the detection of butyryl-coenzyme A (CoA) transferase [28] and butyrate-kinase [29] genes. Genes encoding for *Clostridium difficile* toxin B [30] and virulence factors of *Escherichia coli* [31] were also measured. Primer pairs for bacterial groups, genes involved in the butyrate production and toxins are shown in Table 1. The copy numbers of virulence factors of *E. coli*, heat-sable (STa and STb) and heat-labile (LT) enterotoxins, and the gene encoding the enteroaggregative heat stable toxin 1 (EAST1), and genes encoding *C. perfringens* α toxin were below the detection limit of 10^4^ gene copies/g in all samples.

**Table 1:** Primers used in the study.

Target bacterial group/gene	Oligonucleotide sequence (5′→ 3′)	Annealing temp (°C)	Productsize (bp)	Reference or source
Universal bacterial primers	HDA1: GC clamp-ACTCCTACGGGAGGCAGCAGT* HDA2: GTATTACCGCGGCTGCTGGCAC	52	200	[53]
Total bacteria	F: CGGYCCAGACTCCTACGGG R: TTACCGCGGCTGCTGGCAC	63	200	[54]
*Bacteroides*-*Prevotella*-*Porphyromonas* group	F: GGTGTCGGCTTAAGTGCCAT R: CGGAYGTAAGGGCCGTGC	60	140	[55]
*Lactobacillus* group	F: AGCAGTAGGGAATCTTCCA R: CACCGCTACACATGGAG	63	341	[56], [57]
*Bifidobacterium* spp.	F: TCGCGTCYGGTGTGAAAG R: CCACATCCAGCRTCCAC	60	243	[55]
*Clostridium* cluster I	F: GTGAAATGCGTAGAGATTAGGAA R: GATYYGCGATTACTAGYAACTC	58	665	[58]
*Clostridium* perfringens α toxin	F: GCTAATGTTACTGCCGTTGA R: CCTCATTAGTTTTGCAACC 6FAM-GCGCAGGACATGTTAAGTTTG-TAMRA	55	109	[59]
*Clostridium* cluster IV	F: GCACAAGCAGTGGAGT R: CTTCCTCCGTTTTGTCAA	60	239	[60]
*Clostridium* cluster XIVa	F: AAATGACGGTACCTGACTAA R: CTTTGAGTTTCATTCTTGCGAA	58	438-441	[60]
Butyryl-CoA transferase	F: GCIGAICATTTCACIGGAAYWSITGGCAYATG R: CCTGCCTTTGCAATRTCIACRAANGC	55	530	[28]
Butyrate-kinase	F: GTATAGATTACTIRYIATHAAYCCNGG R: CAAGCTCRTCIACIACIACNGGRTCNAC	55	301	[29]
*Clostridium* cluster XI	F: ACGCTACTTGAGGAGGA R: GAGCCGTAGCCTTTCACT FAM-GTGCCAGCAGCCGCGGTAATACG-BHQ	58	139	[61]
*Clostridium difficile* toxin B	F: GAAAGTCCAAGTTTACGCTCAAT R: GCTGCACCTAAACTTACACCA FAM-ACAGATGCAGCCAAAGTTGTTGAATT-TAMRA	58	177	[30]
*Enterobacteriaceae* family	F: CATTGACGTTACCCGCAGAAGAAGC R: CTCTACGAGACTCAAGCTTGC	63	195	[62]
STa	F: ATGAAAAAGCTAATGTTGGC R: TACAACAAAGTTCACAGCAG	56	193	[31]
STb	F: AATATCGCATTTCTTCTTGC R: GCATCCTTTTGCTGCAAC	56	204	[31]
LT	F: CTATTACAGAACTATGTTCGG R: TACTGATTGCCGCAATTG	56	291	[31]
EAST1	F: TGCCATCAACACAGTATATCC R: GCGAGTGACGGCTTTGT	56	109	[31]

F, forward primer; R, reverse primer.

*GC clamp sequence – CGCCCGGGGCGCGCCCCGTGGCGGGGCGGGGGCGCGCGGGG.

### Analysis of cecal microbiota profiles by PCR-DGGE

The V3 region of 16S rDNA was amplified using the primer pairs HDA1-GC and HDA2 (Table 1). Amplicons were used as templates in a second PCR with the same primer pairs. DGGE analysis was performed using DCode Universal Mutation Detection System (Bio-Rad) on a 6% (w/v) polyacrylamide gel (37.5∶1 acrylamide:bisacrylamaide). DGGE gels contained a denaturing gradient from 30 to 55% of 7 M urea and 40% (w/v) formamide. Electrophoresis was carried out in 1x Tris-acetate-EDTA (TAE) buffer (40 mM Tris-base; 20 mM acetic acid; 1 mM EDTA; pH = 8.5) for 4 h at a constant voltage of 130 V and a temperature of 60°C. Staining was done in SYBR Safe 1x solution (Invitrogen) for 1 h and gels were visualized by UV illumination. Profile analysis of DGGE patterns was conducted using BioNumerics software (version 6.01, Applied Maths, Belgium) based on Pearson correlation coefficient (curved based) and unweighted-pair group method using arithmetic averages (UPGMA). A reference sample was run on each gel and used for normalization between the gels.

### Short-chain fatty acids (SCFA) quantification in cecal contents

Short chain fatty acids (SCFA) were quantified in the cecum, where highly fermentable oligosaccharides are fermented in the rodent intestine [32]. Cecal content was weighted and mixed with 3 volumes of 25% phosphoric acid containing 100 mmol/L iso-caproate as internal standard, and centrifuged at 17 000 g for 10 min. The concentration of SCFA in the supernatant was determined by a gas chromatography on a Stabilwax DA column (30 m, 0.53 mm ID, 0.5 µm film thickness) (Restek, Bellefonte, PA, USA) as previously described [33]. External standards were used to determine the concentration of acetate, propionate, butyrate, iso-butyrate, valerate and iso-valerate and results were expressed as µmol SCFA/g of cecal content.

### Statistical analysis

Statistical analysis of data was carried out with the Statistical Analysis Systems (SAS Institute, Inc.). Differences between diets were evaluated by two-way analysis of variance followed by comparison of all possible pair-wise comparisons between means of different groups. Bonferroni adjustment of the alpha level was used to correct for multiple comparisons and a probability of <0.05 was considered statistically significant. All results were presented as means ± SEM. Spearman's correlation test was employed to check for any correlation between mucosal inflammation and SCFA using GraphPad Prism version 5.00 (GraphPad Software). In addition, linear discriminant analysis and principle component analysis (PCA) were performed using JMP software (version 9.0.1, SAS Institute Inc., NC) to examine correlations between gene copies of bacterial groups, SCFA and intestinal inflammation.

## Results

### Effects of diets and fibres on intestinal inflammation

**Figure 1:**
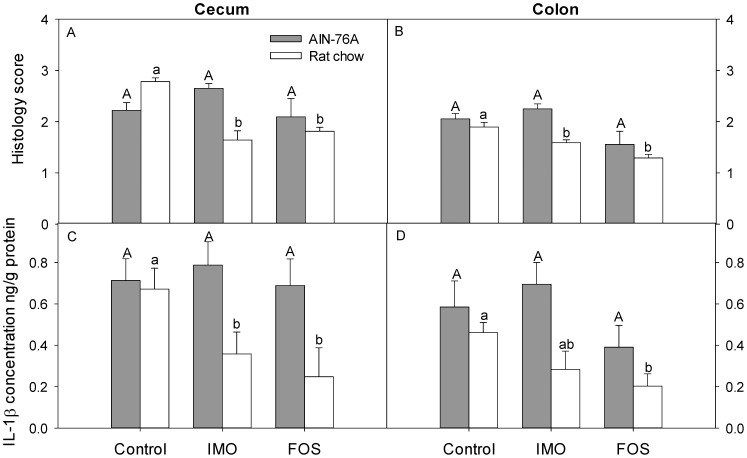
Effect of diet in intestinal inflammation. Inflammation was assessed by histological scoring of inflammation in cecum (panel A) and colon (panel B), and by quantification of the IL-1β in cecal (panel C) and colonic (panel D) tissues collected from HLA-B27 transgenic rats fed AIN-76A or rat chow diet supplemented either with FOS or IMO, or not supplemented. Values are expressed as means ± SEM, n = 6 and n = 8 for rat chow and AIN-76A diet, respectively. Values in the same panel that do not share a common superscript differ significantly (*P*<0.05, Bonferroni adjustment).

Supplementation of rat chow with either FOS or IMO significantly reduced cecal and colonic microscopic inflammation compared to control animals (Figure 1). In contrast, supplementation of the same oligosaccharides to AIN-76A diet did not reduce cecal and colonic inflammation (Figure 1). Reduced inflammation upon supplementation of rat chow with IMO or FOS was associated with reduced concentrations of the pro-inflammatory cytokine IL-1β (Figure 1). FOS or IMO thus reduced intestinal inflammation when supplemented to rat chow, but had no effect when added to AIN-76A diets.

### Analysis of intestinal microbiota by quantitative PCR

To determine whether the different diets and fibres had divergent effects on intestinal microbiota, major bacterial groups in cecal digesta were quantified by PCR (Table 2). In HLA-B17 transgenic rats, the cecum is the site with the most significant inflammation [25], [26]. Supplementation with FOS treatment significantly increased copy numbers of *Bifidobacterium* spp. and *Enterobacteriaceae* family; copy numbers of *Clostridium* cluster I and IV were decreased compared to the control and IMO groups for both diets (Table 2). With rat chow diet, gene copy numbers of butyrate-kinase and *Clostridium* cluster XI were higher in FOS-fed animals compared to control and IMO treatments. Supplementation of feed with IMO induced only few significant changes of cecal microbiota. Notably, IMO supplementation decreased the abundance of *Enterobacteriaceae* in rats fed rat chow, and decreased the gene copy numbers of the *Clostridium difficile* toxin B in rats fed AIN76A. *C. difficile* toxin B was detected only in samples collected from animals on rat chow (Table 2).

**Table 2:** Quantification of predominant bacterial groups and bacterial genes in cecal contents of HLA-B27 transgenic rats on AIN-76A diet or rat chow diet at the end of the experimental treatment period (16 weeks of age).

Target bacterial group or gene*		AIN-76A diet			Rat chow diet	
	Control	IMO	FOS	Control	IMO	FOS
*Bacteroides* group	30.6±3.0^A^	29.5±4.0^A^	35.9±6.1^A^	34.0±5.7^a^	39.3±4.0^a^	46.6±3.5^a^
*Lactobacillus* group	1.9±0.8^A^	2.5±0.6^A^	2.8±1.2^A^	9.6±2.9^a^	5.1±2.1^a^	9.9±5.2^a^
*Bifidobacterium* spp.	0.1±0.03^A^	0.2±0.04^A^	2.8±0.7^B^	0.04±0.01^a^	0.08±0.02^a^	3.1±1.0^b^
*Enterobacteriaceae* family	1.2±1.1^A^	0.3±0.2^B^	2.7±1.4^C^	0.009±0.002^a^	0.06±0.03^a^	0.7±0.6^b^
*Clostridium* cluster I	0.4±0.1^A^	0.3±0.1^A^	0.2±0.1^B^	0.2±0.02^a^	0.3±0.1^a^	0.04±0.01^b^
*Clostridium* cluster IV	5.7±1.0^A^	7.9±1.1^A^	0.6±0.1^B^	9.2±1.9^a^	11.9±5.2^a^	2.2±0.6^b^
*Clostridium* cluster XIVa	4.2±1.0^A^	3.0±0.3^A^	2.9±0.9^A^	12.8±1.7^a^	10.3±1.4^a^	11.9±2.9^a^
Butyryl-CoA transferase	0.01±0.003^A^	0.02±0.007^A^	0.02±0.01^A^	0.01±0.003^a^	0.004±0.001^a^	0.01±0.002^a^
Butyrate-kinase	0.01±0.002^A^	0.01±0.002^A^	0.01±0.01^A^	0.03±0.01^a^	0.03±0.01^a^	0.07±0.01^b^
*Clostridium* cluster XI	0.8×10^−5^ ±0.2×10^−5A^	0.9×10^−5^ ±0.4×10^−5A^	0.8×10^−5^ ±0.1×10^−5A^	0.4×10^−4^ ±0.3×10^−4A^	0.0004±0.0003^A^	0.001 ±0.0003^B^
*C. difficile* toxin B	ND**	ND	ND	0.8×10^−4^ ±0.4×10^−4A^	0.02±0.009^B^	0.09±0.05^B^

*Results are represented as means±SEM and depict the percent of the respective bacterial group/gene related to total bacteria. Values obtained with the same primer pair that do not share a common superscript are significantly different (*P*<0.05, Bonferroni adjustment).

**ND, not detected.

**Figure 2:**
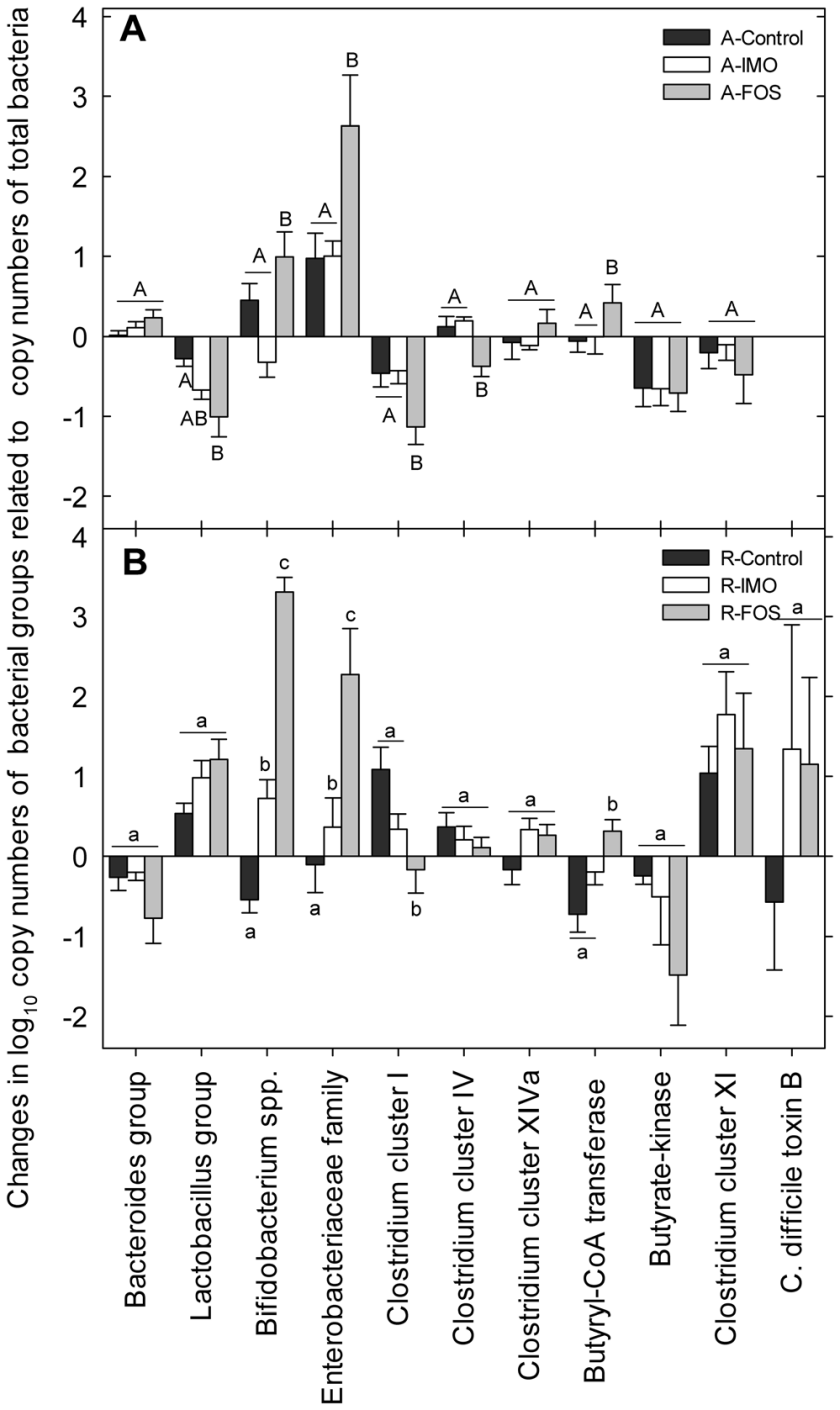
Effects of diets and fibres on fecal bacterial composition. Changes of fecal bacterial composition (week 16 – week 4) in HLA-B27 transgenic rats was evaluated by qPCR. AIN-76A diet is shown in panel A, whereas panel B depicts the results for rat chow diet. Vertical bars represent means and standard error of the mean. Values obtained with the same primer pair that do not share a common superscript are significantly different (*P*<0.05, Bonferroni adjustment).

Changes of intestinal microbiota in the same animal over time were assessed by analysis of fecal microbiota (Figure 2). The supplementation with FOS significantly increased bifidobacteria, *Enterobacteriaceae* and butyryl-CoA transferase genes and decreased the *Clostridium* cluster I when compared to control and IMO groups on both diets. FOS decreased abundance of *Lactobacillus* group and *Clostridium* cluster IV in animals fed AIN-76A (Figure 2).

### Qualitative analysis of DGGE band profiles of cecal microbiota

**Figure 3:**
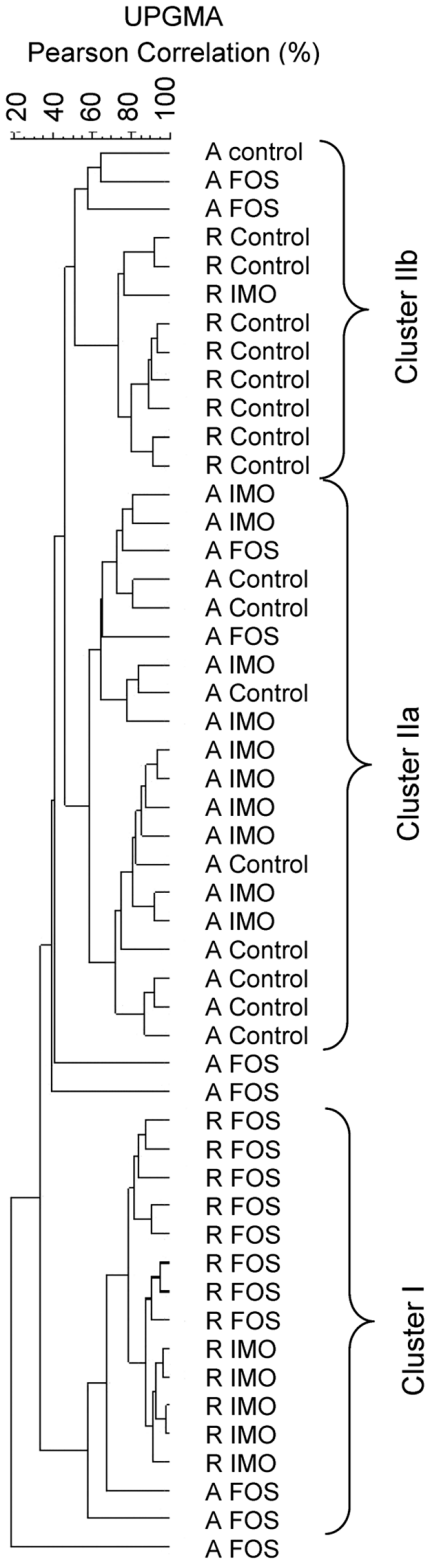
PCR-DGGE analysis of cecal microbiota of HLA-B27 transgenic rats. Samples are labeled with animal number, diet, and oligosaccharide preparation used. Animals on the AIN-76A diet are indicated with A; animals on the rat chow diet are indicated with R. Diets were supplemented with fructo-oligosaccharides (FOS) or with isomalto-oligosaccharides (IMO), or not supplemented with oligosaccharides (control animals). Cecal contents were collected for DNA extraction at the end point of the fibre treatment (16 weeks of age). UPGMA arithmetic algorithm based on Pearson correlation coefficient was used to conduct the dendrogram.

Effects of diets and fibres on qualitative changes of the composition of cecal microbiota in HLA-B27 transgenic rats were determined by PCR-DGGE. Cluster analysis revealed that the DGGE profiles were separated into two main clusters (Figure 3). Cluster I consisted of FOS- and IMO-fed animals on rat chow diet, which also showed reduced cecal and colonic inflammation (Figure 1). FOS- and IMO-fed animals on rat chow clustered separately, demonstrating differences in cecal microbiota composition between FOS and IMO dietary interventions. Cluster II consisted of inflamed animals and comprised the majority of animals fed AIN-76A (Cluster IIa). Animals fed rat chow without oligosaccharide supplementation formed a distinct group in the Cluster II (Cluster IIb) (Figure 3). FOS-fed rats on AIN-76A were spread throughout the whole dendrogram.

### SCFA composition of cecal contents

**Figure 4:**
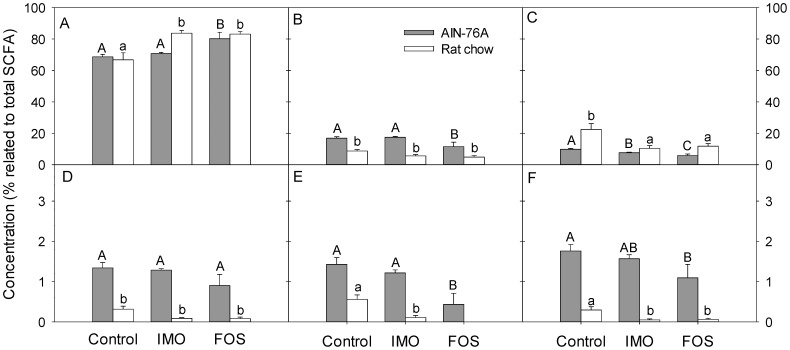
Effects of diets and fibre additives on SCFA concentration in cecal contents. Samples were collected at the end point of the fibre treatment of HLA-B27 transgenic rats. Vertical bars show concentration of the respective SCFA related to the concentration of total SCFA and values are expressed as means ± SEM, n = 6 and n = 8 for rat chow and AIN-76A diet, respectively. Values that do not share a common superscript differ significantly (*P*<0.05, Bonferroni adjustment). A – acetate; B – propionate; C – butyrate; D – isobutyrate; E – valerate; F – isovalerate. Total SCFA concentrations were 220±23, 239±11 and 183±17 µmol/g for control, IMO, and FOS groups, respectively in digesta from treatment groups on rat chow and 118±11, 195±17 and 132±10 µmol/g for control, IMO, and FOS groups, respectively, in digesta from treatment groups on AIN-76.

To assess the effect of diet and fibre supplementation on production of microbial metabolites, SCFA were quantified in the cecum. Total SCFA concentrations (220±23, 239±11 and 183±17 µmol/g, for control, IMO, and FOS groups, respectively) in digesta from treatment groups on rat chow did not differ significantly between each other. However, supplementation of AIN-76A with IMO resulted in significantly higher SCFA concentrations (195±17 µmol/g) in comparison with the control and FOS groups (118±11 and 132±10 µmol/g; *P* = 0.001 and *P* = 0.0001, respectively). The relative concentration of acetate was significantly increased in IMO- and FOS-treated rats versus control group on rat chow (Figure 4A). FOS supplementation also increased relative acetate concentrations in rats fed AIN-76A (Figure 4A). Proportions of propionate and iso-butyrate showed little variation between diets (Fig. 4B and 4D). Valerate and isovalerate were reduced in comparison with control animals when FOS was included in the AIN-76A diet (Figure 4E and 4F).

### Correlation of SCFA concentrations to microbiota and inflammation

Spearman's correlation analysis was performed for cecal parameters to detect associations of bacterial metabolites, the abundance of bacterial groups and intestinal inflammation. Significant correlations are shown in Table 3. The relative concentration of acetate was negatively correlated to the cecal histology score and the levels of IL-1β. Interestingly, the relative concentrations of propionate and all branched chain fatty acids were positively correlated with inflammation markers (Table 3). Butyrate was positively correlated with clostridial clusters IV and XIVa. The clostridial cluster XIVa and the *Lactobacillus* group showed negative correlations with propionate, iso-butyrate or iso-valerate. The *Bacteroides-Prevotella-Porphyromonas* group was positively associated with acetate and negatively correlated with iso-butyrate and valerate (Table 3).

**Table 3:** Correlations between the cecal parameters SCFA concentration, inflammation markers, and copy numbers of bacterial taxa.

SCFA/Bacterial group	Acetate	Propionate	Butyrate	Iso-butyrate	Valerate	Iso-valerate
*Bacteroides* group	**0.44 (0.001)**	−0.34 (0.016)		−0.33 (0.019)	**–0.43 (0.002)**	−0.35 (0.013)
*Lactobacillus* group	0.26 (0.07)	**−0.56 (<0.001)**		**−0.55 (<0.001)**	−0.36 (0.009)	**−0.54 (<0.001)**
*Bifidobacterium* spp.	0.35 (0.012)		−0.37 (0.007)		−0.30 (0.034)	
*Enterobacteriaceae* family			−0.30 (0.035)			
*Clostridium* cluster I	−0.30 (0.032)	0.34 (0.014)		0.31 (0.025)	**0.49 (<0.001)**	0.32 (0.023)
*Clostridium* cluster IV	**−0.44 (0.007)**		**0.41 (0.003)**		**0.42 (0.004)**	
*Clostridium* cluster XIVa		**−0.47 (<0.001)**	**0.57 (<0.001)**	**−0.45 (0.001)**	−0.36 (0.01)	**−0.43 (0.002)**
IL-1 β concentration (pg/mg protein)	**−0.44 (0.001)**	**0.47 (0.004)**		**0.54 (0.001)**	**0.45 (0.001)**	**0.54 (0.001)**
Histology score	**−0.56 (<0.001)**	**0.44 (0.001)**		**0.48 (<0.001)**	**0.40 (0.004)**	**0.43 (0.002)**

Correlation coefficients were assessed by Spearman's correlation test, P-value are shown in brackets. Values are shown only for significant correlations between variables (*P*<0.05). Correlations with r>0.4 are indicated in bold face type.

### Multivariate data analysis

The relationship between dietary intervention and intestinal microbiota was further assessed using linear discriminant analysis (Figures S1A and S1B in File S1). Cecal and fecal samples from FOS-treated rats clustered separately from IMO-treated and control rats for both diets. Animals on rat chow clustered separately from animals on AIN-76A. IMO-treated animals and control animals on AIN-76A clustered together but IMO-treated animals and control animals on rat chow were separated into two clusters. Linear discriminant analysis thus supports the dendrogram obtained by DGGE (Figure 3).

**Figure 5:**
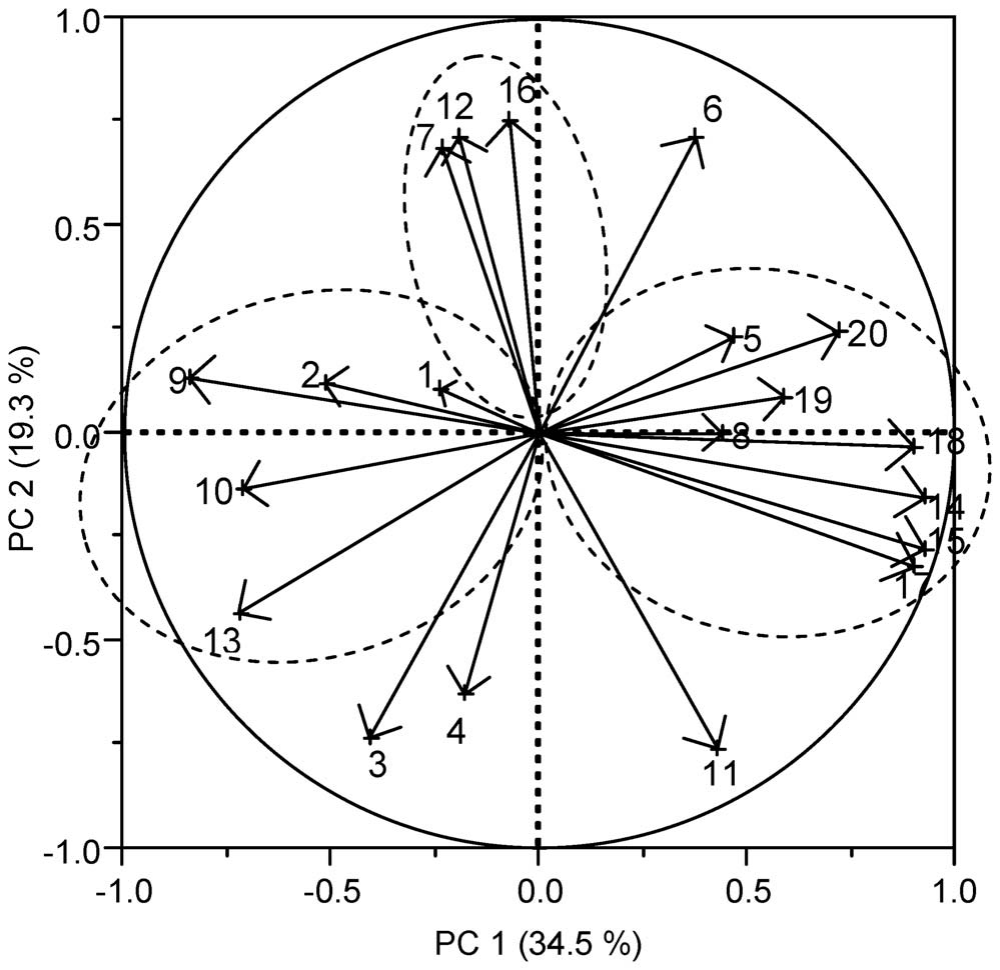
Principle component analysis (PCA). Loading plot of the first two principle components (PC1 and PC2) was build based on the following variables in cecum: gene copy numbers of bacterial groups; SCFA, histology score and mucosal IL-1β concentration. The loading plot of the first two principle components (PC1 and PC2) depicts the correlations between the different variables. Numbers represent individual variables as follows: 1, *Bacteroides* group; 2, *Lactobacillus* group; 3, *Bifidobacterium* spp.; 4, *Enterobacteriaceae* family; 5, *Clostridium* cluster I; 6, *Clostridium* cluster IV; 7, *Clostridium* cluster XIVa; 8, Butyryl-CoA transferase gene; 9, Butyrate-kinase gene; 10, *Clostridium* cluster XI; 11, *Clostridium difficile* toxin B; 12, total SCFA; 13, acetate; 14, propionate; 15, iso-butyrate; 16, butyrate; 17, iso-valerate; 18, valerate; 19, cecal IL-1β concentration; 20, histology score of cecum.

Principle component analysis (PCA) of the abundance of bacterial groups, bacterial metabolites and inflammation markers is shown in Figure 5. The loading plot of the first two eigenvalues (PC 1 and PC 2) separates the variables into three main clusters. *Clostridium* cluster I, butyryl-CoA transferase gene, propionate, iso-butyrate, valerate and iso-valerate, together with the inflammation markers, histology score and levels of IL-1β, clustered into one group located on the right quadrant of the graph and were highly influenced by PC 1. A second cluster, containing *Bacteroides* and *Lactobacillus* groups, acetate, butyrate-kinase gene and clostridial cluster XI, was situated opposite to the first cluster, representing a negative correlation. *Clostridium* cluster XIVa, total SCFA and butyrate formed a third cluster located between the two mentioned above, and were influenced positively by PC 2 (Figure 5).

## Discussion

Diet and dietary fibres can restore dysbiosis resulting from intestinal inflammation [8], [10], [11], [17] and potentially reduce intestinal inflammation in IBD. Animal studies demonstrated that FOS reduced experimental colitis [11], [15]; however, their beneficial effect depends on the dietary background [17]. Small scale trials in active UC indicated protective effects of FOS administration [34] but FOS were not effective in treatment of active Crohn's disease [35]. The divergent outcomes of IBD-intervention studies with FOS in humans and animals may reflect the lack of information on their mode of action, and the lack of knowledge on their interaction with other components of the diet.

Beneficial effects of FOS, including their potential benefits in prevention or treatment of IBD, were attributed to their ability to stimulate commensal bifidobacteria [11], [14], [15]. This study compared the effect of bifidogenic FOS with the effect of IMO, which exhibit no bifidogenic effects in rats [21]. Moreover, oligosaccharides were added to rat chow and a purified diet, AIN-76A, in a factorial experimental design. As outlined below, the comparison across these dietary interventions indicates that the specific effect of FOS and IMO on the composition of intestinal microbiota at the species level and their protective effect in IBD are independent of each other.

The present study demonstrated that IMO may reduce chronic intestinal inflammation in a colitis model. In contrast to previous studies in healthy rodents [21], IMO induced only minor shifts in intestinal microbiota in the rodent colitis model, reflecting the influence of intestinal inflammation on intestinal microbiota [4], [5] that was also observed by DGGE-fingerprinting. However, IMO supplementation significantly increased cecal microbial metabolic activity. Intestinal production of SCFA, particularly butyrate, is recognized as an important modulator of gut immunity which makes a strong contribution to the tolerance of commensal microbiota [36].

In keeping with the prior observations, dietary supplementation with FOS increased the abundance of intestinal bifidobacteria and *Enterobacteriaceae* [11], [14], . This effect was observed in animals receiving FOS -supplemented raw chow as well as FOS-supplemented AIN-76A. FOS addition to AIN-76A, however, did not reduce cecal inflammation. Conversely, IMO supplementation of rat chow prevented colitis without bifidogenic effect. Taken together, these data suggest that specific changes in the composition of intestinal microbiota that are induced by oligosaccharide supplementation do not mediate colitis reduction.

Non-digestible carbohydrates are fermented by intestinal bacteria to SCFA in the cecum and colon. Acetate, propionate and butyrate are the major end-products of fermentation [37]. IBD patients exhibited reduced colonic SCFA concentrations [38]. In the present study, total cecal SCFA were higher in animals on rat chow when compared to animals on AIN-76 but the relative concentration of branched chain SCFA in rats fed AIN-76A was more than twofold higher when compared to rats fed rat chow. Branched chain SCFA are exclusively derived from amino acid fermentation by strict anaerobes [39], [40]. The higher linear SCFA levels in animals fed rat chow thus reflect enhanced intestinal carbohydrate fermentation based on the higher content of non-digestible but fermentable carbohydrates in rat chow when compared to AIN-76A. Total SCFA concentrations, relative butyrate concentrations, and the abundance of *Clostridium* clusters IV and XIVa were correlated. The major fibre-fermenting and butyrate-producing bacterial species in the large intestine belong to those two clostridial clusters [41]. The effect of the diet on cecal SCFA formation was also supported by the quantification of genes encoding for butyryl-CoA transferase and butyrate-kinase.

PCA and correlation analysis consistently indicated that markers of inflammation were negatively correlated to the relative concentration of cecal acetate, and positively correlated to the relative concentrations of cecal branched chain SCFA. PCA and correlation analysis thus suggest that inflammation was associated with reduced carbohydrate fermentation and increased protein fermentation in the large intestine. This association of branched-chain fatty acids and inflammation was also reported when intestinal inflammation was induced by environmental particulate matter [42]. In contrast, microbial production of linear SCFA in the large intestine was proposed to exhibit anti-inflammatory effects due to the ability of SCFA to suppress NF-κB reporter activity and the release of pro-inflammatory cytokines [43], [44]. Butyrate also plays an important role as major energy source for colonocytes [45], [46]. Intestinal SCFA may contribute to the anti-inflammatory effects of dietary fibres in the treatment of IBD [11], [43].

It was recently reported that feeding purified diets enhanced the susceptibility of mice to DSS-induced colitis; this effect was partially mediated by the gut microbiome and was associated with diet-mediated effects on the systemic immune response [47]. Our study demonstrated that the effect of the overall dietary fibre content on intestinal microbiota and cecal SCFA formation in HLA-B27 transgenic rats was greater than the effect of supplementation with IMO or FOS. It is noteworthy that studies in two different animal models for IBD provided comparable results related to the effect of FOS in purified diets ([16], [17], this study). Colitis in HLA-B17 transgenic rats is induced by activation of INF-γ-producing CD4^+^ T cells [15], [26] whereas the chemically induced colitis in the DSS model [16], [17] is characterized by a Th1/Th2 response. The AIN76A diet is highly digestible, offers sucrose as main dietary carbohydrate and its sole source of fibre is cellulose, which is not fermented by human or rodent gut microbiota [48]. Rat chow consists of natural sources that include diverse fibres, fatty acids, and phytochemicals, although their composition is poorly defined and may not be consistent over time. High sucrose consumption and a high intake of refined carbohydrates have been associated with an increased risk of IBD [7], [49]. Comparison of diets abundant in whole grains versus diets with high refined grains demonstrated that an increased bacterial diversity, a higher *Firmicutes/Bacteroides* ratio, increased the abundance of the *Lactobacillus* group, and immunological improvements upon inclusion of whole grains in the diet [50], [51]. The effect of whole grains on gut microbes was attributed to the diverse fibre components in whole grains that are absent in refined grains or purified dietary fibre supplements [51]. Phytochemicals such as phenolic compounds may additionally contribute to the beneficial effects of whole grains on host health. Phenolic compounds in apples reduced colitis in the same rat colitis model [52].

In conclusion, this study indicates that a variety of dietary non-digestible oligosaccharide can mediate protection against intestinal inflammation. Protective effects of diet or supplementation with non-digestible oligosaccharides are not related to the stimulation of specific bacterial groups or genera but relate to diet effects on the metabolic activity of intestinal microbiota. Specifically, intestinal inflammation correlated to reduced carbohydrate fermentation in the large intestine and a higher contribution of amino acid fermentation to intestinal microbial metabolism. This study suggests that colitis is associated with increased formation of branched-chain SCFA and supports earlier observations that successful (dietary) intervention in IBD is associated with increased carbohydrate fermentation in the large intestine, and increased intestinal straight-chain SCFA [11], [16], [44], [45]. The role of other components of whole-grain diets, particularly unsaturated fatty acids and phenolic compounds [10], [52] in prevention of IBD remains to be elucidated.

## Supporting Information

File S1 **Figure S1. Linear discriminant analysis for cecal (Panel A) and fecal (Panel B) samples.** Analysis was performed based on gene copy numbers of bacterial groups. HLA-B27 transgenic rats were fed a AIN-76A diet (indicated with A) or a rat chow diet (indicated with R). Diets were supplemented with fructo-oligosaccharides (FOS; □, ▪) or with isomalto-oligosaccharides (IMO; Δ, ▴), or not supplemented with oligosaccharides (control; ○, •) for a period of 12 weeks. Each symbol represents an individual animal. Numbers represent variables discriminating between the groups as follows: 1, *Bacteroides* group; 2, *Lactobacillus* group; 3, *Bifidobacterium* spp.; 4, *Enterobacteriaceae* family; 5, *Clostridium* cluster I; 6, *Clostridium* cluster IV; 7, *Clostridium* cluster XIVa; 8, Butyryl-CoA transferase gene; 9, Butyrate-kinase gene; 10, *Clostridium* cluster XI; 11, *Clostridium difficile* toxin B. **Table S1. Basic composition of chemically defined diet (AIN-76A).** **Table S2. Chemical composition of rat chow diet (5053 PicoLab Rodent Diet 20).**
(PDF)
