# Supplementary material for: Chemically Defined Diet Alters the Protective Properties of Fructo-Oligosaccharides and Isomalto-Oligosaccharides in HLA-B27 Transgenic Rats
Source: PLoS One. 2014 Nov 4;9(11):e111717. doi: 10.1371/journal.pone.0111717 (PMC4219767; doi:10.1371/journal.pone.0111717)

Online Supplemental Material.

Online Supplemental Table 1. Basic composition of chemically defined diet (AIN-76A).

**Ingredients**	**per 100 g**
Casein	20.0
DL-Methionine	0.3
Sucrose	49.5
Corn Starch	15
Corn Oil	5
Cellulose	5
Mineral Mix	3.5
Vitamin Mix	1.5
Choline Bitartrate	0.22
Vitamin K, MSB complex	0.0003
Ethoxyquin, antioxidant	0.001

*Purchased from Teklad Custom Research Diet (Harland Laboratories, Madison, WI, USA).

Online Supplemental Material.

Online Supplemental Table 2. Chemical composition of rat chow diet (5053 PicoLab® Rodent Diet 20).

Ingredients	per 100 g
Crude protein, g	20.0
Fat (ether extract), g	5.0
Fat (acid hydrolysis), g	5.6
Crude fibre, g	4.7
Nitrogen-free extract, g	52.9
Total digestible nutrients, g	76.2
Minerals	
Ash, g	6.1
Calcium, g	0.81
Phosphorus, g	0.63
Phosphorus (non-phytate), g	0.33
Potassium, g	1.07
Magnesium, g	0.22
Sulfur, g	0.34
Sodium, g	0.30
Chloride, g	0.51
Fluorine, ppm	10
Iron, ppm	220
Zinc, ppm	87
Manganese, ppm	85
Copper, ppm	13
Cobalt, ppm	0.71
Iodine, ppm	0.97
Chromium, ppm	0.81
Selenium, ppm	0.30
Vitamins	
Carotene, ppm	1.5
Vitamin K (as menadione), ppm	3.3
Thiamin hydrochloride, ppm	17
Riboflavin, ppm	8.0
Niacin, ppm	90
Pantothenic acid, ppm	17
Choline chloride, ppm	2000
Folic acid, ppm	3.0
Pyridoxine, ppm	9.6
Biotin, ppm	0.30
B_{12}, mcg/kg	51
Vitamin A, IU/gm	15
Vitamin D_3, IU/gm	2.2
Vitamin E, IU/gm	99

*Purchased from Lab Diet Inc., Leduc, AB, Canada. Nutrient content is obtained from http://www.labdiet.com/pdf/5053.pdf.

Online Supplemental Figure S1. Linear discriminant analysis for cecal (Panel A) and fecal (Panel B) samples. Analysis was performed based on gene copy numbers of bacterial groups. HLA-B27 transgenic rats were fed a AIN-76A diet (indicated with A) or a rat chow diet (indicated with R). Diets were supplemented with fructo-oligosaccharides (FOS; □, ■) or with isomalto-oligosaccharides (IMO; Δ, ▲), or not supplemented with oligosaccharides (control; ○, ●) for a period of 12 weeks. Each symbol represents an individual animal. Numbers represent variables discriminating between the groups as follows: 1, *Bacteroides* group; 2, *Lactobacillus* group; 3, *Bifidobacterium* spp.; 4, *Enterobacteriaceae* family; 5, *Clostridium* cluster I; 6, *Clostridium* cluster IV; 7, *Clostridium* cluster XIVa; 8, Butyryl-CoA transferase gene; 9, Butyrate-kinase gene; 10, *Clostridium* cluster XI; 11, *Clostridium difficile* toxin B.

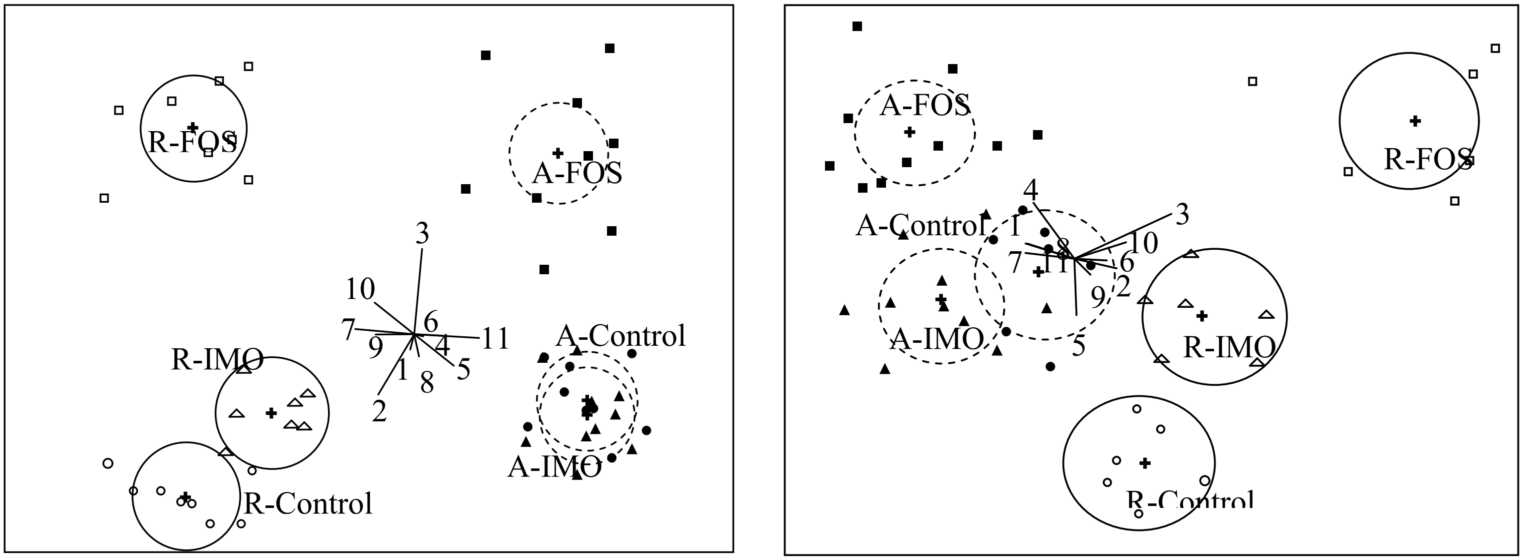

Supplement: File S1 — Figure S1. Linear discriminant analysis for cecal (Panel A) and fecal (Panel B) samples. Analysis was performed based on gene copy numbers of bacterial groups. HLA-B27 transgenic rats were fed a AIN-76A diet (indicated with A) or a rat chow diet (indicated with R). Diets were supplemented with fructo-oligosaccharides (FOS; □, ▪) or with isomalto-oligosaccharides (IMO; Δ, ▴), or not supplemented with oligosaccharides (control; ○, •) for a period of 12 weeks. Each symbol represents an individual animal. Numbers represent variables discriminating between the groups as follows: 1, Bacteroides group; 2, Lactobacillus group; 3, Bifidobacterium spp.; 4, Enterobacteriaceae family; 5, Clostridium cluster I; 6, Clostridium cluster IV; 7, Clostridium cluster XIVa; 8, Butyryl-CoA transferase gene; 9, Butyrate-kinase gene; 10, Clostridium cluster XI; 11, Clostridium difficile toxin B. Table S1. Basic composition of chemically defined diet (AIN-76A). Table S2. Chemical composition of rat chow diet (5053 PicoLab Rodent Diet 20). (PDF) [file pone.0111717.s001.pdf]
